# Hernioplasty with Peritoneal Flap for the Surgical Treatment of Umbilical Hernia in Swine

**DOI:** 10.3390/ani12233240

**Published:** 2022-11-22

**Authors:** Filippo Spadola, Veronica Cristina Neve, Claudia Dina Interlandi, Andrea Spadaro, Francesco Macrì, Nicola Maria Iannelli, Giovanna Lucrezia Costa

**Affiliations:** 1 Department of Veterinary Sciences, University of Messina, Viale Palatucci, 13, 98168 Messina, Italy; 2 Experimental Zooprophylactic Institute of Sicily “A. Mirri”, Via Gino Marinuzzi, 3, 90129 Palermo, Italy; 3 DVM Freelance, Via Risorgimento 6/D, 97015 Modica, Italy

**Keywords:** hernia surgery, hernioplasty, swine surgery, abdominal hernias, omphalocele

## Abstract

**Simple Summary:**

A congenital umbilical hernia was diagnosed in eight swine aged between 4 and 12 weeks, who underwent a clinical examination following the finding of a mass protruding from the umbilical regions. Palpation showed the presence of a fibrous and regular hernial ring allowing the diagnosis of an umbilical hernia. This condition was successfully surgically corrected under general and local anesthesia by associating with traditional herniorrhaphy, a hernioplasty with the use of an autologous peritoneal flap as a prosthesis. The surgeries were successfully conducted, and follow-ups carried out at 7, 30 and 60 days postoperatively showed the absence of recurrence, confirming the complete healing of the lesions and the functional recovery of the herniated organs. This surgical approach has been shown to be highly effective in the treatment of umbilical hernia of swine because it has allowed for providing considerable mechanical resistance to the herniorrhaphy performed, avoiding the use of synthetic prosthetic materials. Synthetic meshes are histocompatible but are foreign bodies that make the surgical wound sensitive to bacterial colonization. The autologous peritoneal flap ensures excellent healing results thanks to its ability to evoke the inflammatory response by promoting the proliferation of blood vessels and the migration of defense and reaction cells for the formation of granulation tissue.

**Abstract:**

Background: Umbilical hernia is one of the most common developmental defects in swine, producing large economic losses for farmers, forced to slaughter animals at a younger age and therefore at a lower weight to prevent fatal complications. This study describes a surgical technique to repair umbilical hernia through the use of autologous prostheses, allowing recovery of the affected animals; Methods: After a general examination of the swine and examination of the lesions, the swine were anesthetized and underwent surgery. The surgery was performed by combining the traditional herniorrhaphy with the inclusion and fixation of a peritoneal flap obtained from the incision of the same hernial sac; Results: Follow-ups were carried out at 7, 30 and 60 days and demonstrated healing in all of the treated subjects; Conclusions: The use of this surgical technique allows for providing resistance to herniorrhaphy performed through the use of a cost-free autologous biomaterial prosthesis, with excellent tissue compatibility. This might allow for reducing significantly the rate of relapses and eliminating the risk of rejection.

## 1. Introduction

An umbilical hernia is one of the most common birth defects found in swine, causing significant economic losses and severe welfare problems. In fact, animals with an umbilical hernia generally have reduced performance, showing low growth rates and low meat quality, but also pain and discomfort that could also cause their death, representing an element of concern where swine farming is widely practiced [1,2].

It has generally been accepted that the etiology of umbilical hernia has a genetic component linked to the presence of a full penetrance recessive gene or an incomplete penetrance dominant gene, the transmission mechanisms of which are still poorly understood [3,4,5,6]. A “familial” (hereditary) cause has been suggested and it has recently been shown by Zhao et al., that some specific genes are associated with this condition but nevertheless do not have absolute control [7,8]. In general, compared to other types of hernias, there appears to be a much higher interaction between genetics and environmental factors for umbilical hernias. The development of hernias is correlated, in fact, to environmental conditions that interfere with the closure of the umbilical cord [9]. Some authors including Ding et al., have reported that an unsanitary delivery environment can lead to a bacterial infection of the umbilical stump, which can potentially result in failure to close or heal the umbilical cord [1,10,11]. Piglets with umbilical cord infection at birth (or shortly thereafter), which show a consequent slowing of umbilical scarring, are more predisposed to the onset of hernia [10]. The umbilical infection, in fact, can cause the weakening of the adjacent abdominal wall and cause an acquired umbilical hernia [12]; this can occur as a local infection or secondary to systemic diseases. Furthermore, other factors that can influence the development of umbilical hernias are abnormal traction of the umbilical cord at birth or any movement that leads to abnormal stretching of the cord (slippery floors, long cord that gets trapped in the grating, long cord on which supports the sow’s foot, etc.); cutting the umbilical cord too close to the abdominal wall [4,13,14,15,16,17]; the sucking of the umbilicus by the other piglets; weakness of the abdominal muscles; nutritional factors.

In 1994, Searcy-Bernal et al., reported that most umbilical hernias appear in swine between the ages of 9 and 14 weeks [1] probably due to the rapid growth of swine during this period which, combined with the increase in weight of the abdominal organs, leads to a significant increase in the size of the hernia. The incidence of umbilical hernias in swine ranges from 0.4% to 1.2% in commercial herds [1,9,10,18] depending on the breed, farm, and production system [1,19] although it has been shown that the frequency of hernias can be as high as 6.7% [20].

But the incidence also varies according to race and sex. In fact, several studies have shown that umbilical hernias are the most common congenital pathologies in Landrace, Large White and Duroc pigs. Regarding sex, the literature is in disagreement between those who affirm a higher risk of developing umbilical hernia in females [21] and those who did not find differences between females and males [9].

Clinically, the umbilical hernia appears as a swelling of variable volume, depending on the size of the hernial ring, with a rounded and regular shape and variable consistency based on the content, in correspondence with the umbilical scar. On palpation, it is not hot and painful, and on deeper palpation of the swelling, it is possible to detect the size of the umbilical ring and the contents of the hernia. Sometimes the intestine or other abdominal structures can be palpated which can be brought back (reduced) within the abdominal cavity; in this case, the hernia will be reducible. If, on the other hand, the umbilical sac is hot and painful, and the contents cannot be reduced due to large adhesions, strangulation or intestinal obstruction must be suspected [22]. In this case, the animals will show typical symptomatology of a colic picture [23].

The clinical alterations depend on the state of the herniated part and range from zero to extremely serious or potentially lethal in case of obstruction of the intestinal lumen. Usually, the general condition of the subjects is good, unless there is secondary impairment to a visceral incarceration in the hernial sac. In these cases, there may be an alteration of the general conditions due to impairment of the functionality of some organs incorporated in the hernia, for mechanical reasons [23]. Occasionally, in fact, a hernia can strangle itself, which occurs when the protruding tissue becomes edematous and is incarcerated. Strangulation cuts off the blood supply and can lead to infection, necrosis and potentially life-threatening conditions [24]. Diagnosis is usually simple and most umbilical hernias are diagnosed on clinical examination by palpation, which highlights the presence of the hernial port and the fibrous hernial ring.

The differential diagnosis should be made with umbilical abscess, hematoma, urachal cyst caused by the persistence of urachus (or patent urachus) and neoplasms.

Umbilical hernia and umbilical abscess are often seen together, especially in cattle and swine. According to Monsang, an exploratory puncture may be required for confirmation in these cases [25]. However, in specific cases, this method is not useful, for example in some chronicized umbilical abscesses in which the walls are so thick, and the pus is so dense that centesis does not provide significant information. In this case, it would be advisable to perform an ultrasound examination to obtain plausible on the content of the swelling and thus be able to establish an adequate therapy [26].

Regarding the treatment, it can be conservative or surgical; in the literature, there are numerous surgical and non-surgical techniques for hernia therapy. Based on the severity of the injury, the appropriate technique should be considered.

The conservative treatment proposed by some authors, such as Pollicino in swine and Greenwood and others in foals, have attempted a non-surgical reduction using Elastrator umbilical forceps. This method may be used in the case of umbilical hernias with a diameter of less than 5 cm, which is fully reducible and in the absence of a history of umbilical infection [27,28]. In the past, daily digital exploration of the hernial port was practiced with the aim of irritating the umbilical ring and causing the formation of adhesions to reduce the width of the hernial port. However, these are cheap and fast methods that are now outdated. Some authors, such as Hall or Knudson, attempted the reduction of very small umbilical hernias in piglets through the topical application of irritating solutions, such as concentrated nitric acid, injected into the hernial sac followed by isolation of the treated animal for about 21 days [29]; or, the application on the umbilicus of a solution of 7% iodine or mercuric iodine which develop an acute inflammatory reaction which often causes the proliferation of connective tissue in sufficient quantity to close the hernia [30].

Conservative treatments are not recommended when the hernial port exceeds 5 cm in diameter [31]. Umbilical hernia surgery is the treatment of choice for the correction of these defects, particularly in the case of umbilical hernias aggravated by complications.

If diagnosis is delayed or treatment is not performed promptly, the aggravation can lead to possible complications such as adhesions and hydrocele of the hernial sac, incarceration, twisting [32] and abscess as reported in goats [33].

The approach is usually directed on the hernial sac and depends on the size of the hernial ring and any complications: in the case of simple hernias, the treatment of choice is by herniorrhaphy, that is, the suture of the hernial ring. It can be performed with the “closed” technique in which the internal hernial sac, i.e., the peritoneum, is not incised but remains intact and placed in the abdominal cavity, in order to reposition the dislocated organs in their normal location [30]; the hernial ring is directly sutured, absolutely without tension to allow the formation of collagen and better wound healing. In cases characterized by adhesions or other complications, the “open” method is preferred, which involves incision and opening of the peritoneal hernial sac.

According to Al-Sobayil and Ahmed, herniorrhaphy is the most suitable procedure for the treatment of many types of hernia but, in the case of complications due to the formation of adhesions of the hernial sac with the viscera or the presence of large umbilical hernias, some authors prefer to perform hernioplasty with implantation of prosthetic material [34,35] in order to perform a tension-free suture and avoid any wound dehiscence. This allows you to avoid the ischemia that is created following the incision of the tissues, allowing the formation of collagen and therefore better scarring and healing [34,35].

Despite the excellent results obtained in the treatment of large hernias, the most frequent complication that leads to implant failure and relapse is surgical site infection [36]. To avoid these complications, the prosthetic material should be inert but strong. Among the synthetic materials, polypropylene has these characteristics and is suitable for use even in the presence of infection and contamination [37]. The prostheses used to reduce the surgical breach and facilitate the reconstruction of the abdominal wall are represented not only by polypropylene or polyester nets but also by alloplastic membranes of Gore-Tex [23].

In our work we performed a traditional herniorrhaphy associated with a hernioplasty on all subjects, using an autologous peritoneal flap as a prosthesis; this has allowed us to provide better mechanical resistance to the suture of the hernial ring, to avoid the use of synthetic prosthetic materials and the related risks of rejection and bacterial colonization, guaranteeing excellent healing results thanks to the ability of the autologous prosthesis to evoke the inflammatory response favoring the proliferation of blood vessels and the migration of defense and reaction cells for the formation of granulation tissue and fibrin.

## 2. Materials and Methods

Hernioplasty with peritoneal flap was applied to 8 swine, 3 males and 5 females, aged between 4 and 12 weeks. Three subjects were Landrace breed while the other five were Large-White crossbreeds, weighing about 35–40 kg. All clinical cases were treated by our team in intensive and non-intensive farms in the southeast of Sicily and in particular in the Ragusa area.

All subjects were placed in pre-operative fasting of 24/48 h, with watering suspended from the previous evening. Before the surgery, the animals underwent a careful clinical and pre-anesthetic examination. A thorough medical history was collected, and a general physical examination and an examination of the lesion were performed.

**Figure 1:**
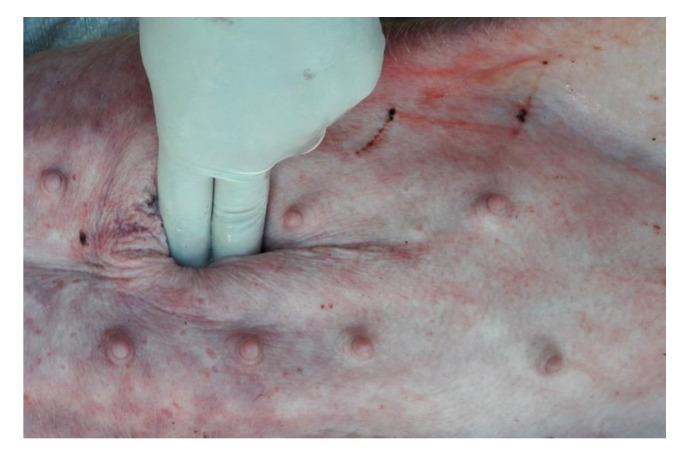
Palpation in dorsal decubitus to reduce the lesion and highlight the hernial port.

The umbilical hernia examination required scrupulous palpation of the umbilical swelling, carried out by placing the animals in dorsal decubitus (Figure 1).

All swine underwent deep sedation and general anesthesia. Each patient received an intramuscular dose of Tiletamine zolazepam, 5 mg/kg (Zoletil 100, Virbac, Carros, France) and Romifidine hydrochloride, 80 µ/kg (Sedivet 1%, Boeringher Ingelheim am Rhein, Germany) administered simultaneously using the same syringe. After achieving general anesthesia, the patients were cannulated in the jugular vein and received a 0.9% NaCl drip at a rate of 5 mL/kg/h, for the entire duration of the operation [38,39].

Analgesia was treated by administering Lidocaine 2%, 2 mg/kg (Lidocaine 2%, Esteve, Girona, Spain). In particular, the Lidocaine 2% was infiltrated both in the surgical planes (skin, muscle plane) and in the hernial sac, by splash.

After sedation, we measured and recorded the heart rate, non-invasive systolic pressure and hemoglobin saturation, using a multi-parametric monitor (AMI Italia srl, Leonardo model, Milan, Italy) and carrying out the count of thoracic excursions in one minute for respiratory rate [40,41]. A 20% increase in the heart rate, respiratory rate and systolic pressure variables compared to the values recorded after sedation, involved the administration of the rescue analgesic represented by boluses of Lidocaine 2% infiltrated in the surgical plans or administered via intraperitoneal splash [39,41,42,43]. In the case of emergence from anesthesia, the administration of half the initial bolus of Tiletamine zolazepam (Zoletil 100, Virbac, Carros, France) and Romifidine hydrochloride (Sedivet 1%, Boeringher Ingelheim am Rhein, Germany) was provided.

Patients were prepared for surgery: positioned in dorsal decubitus and secured with ropes and sandbags. Once the positioning was completed, the preoperative skin preparation was carried out, with the aim of eliminating dirt and surface microorganisms and reducing the resident microbial load to sub-pathogenic levels in a short time and with the least possible tissue irritation. The surgical preparation of the operating field was performed according to art [40,44]: extensive trichotomy of the umbilical region, skin cleansing with disinfectant solutions using alcohol and 10% iodopovidone (Betadine 10%, Viatris healthcare limited, Dublin, Ireland) at least 3 times for each type of disinfectant solution by making circular movements in the centrifugal direction, starting from the center of the trichotomized area towards the periphery, avoiding passing the swab over the areas already disinfected and taking care to change the gauze pads at each step.

The operating field was delimited with drapes positioned at the margins and fixed at the corners with a Backhaus towel clamp. Further manual palpation was performed to highlight the hernial port and, when possible, to bring the herniated organs back into the abdomen.

**Figure 2:**
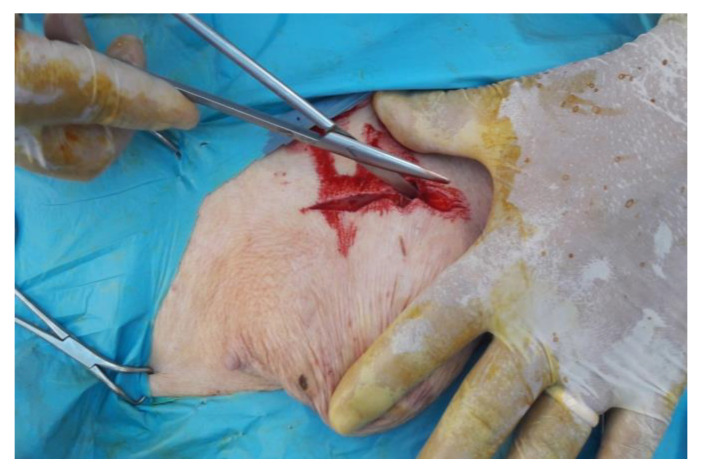
Half-moon shaped skin incision and tissue detachment.

**Figure 3:**
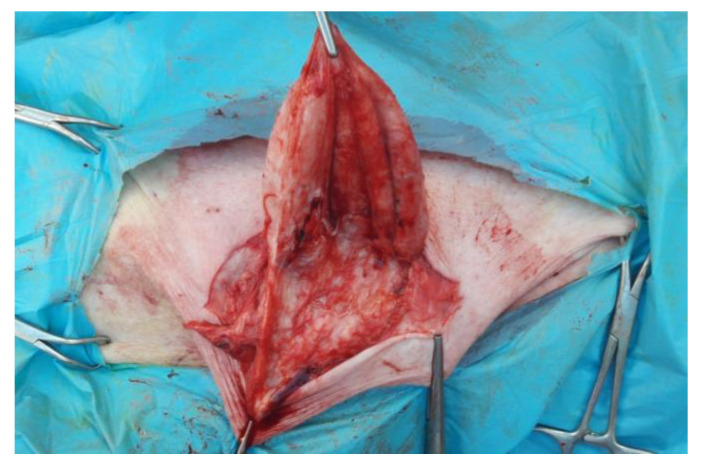
Isolation of the internal hernial sac.

**Figure 4:**
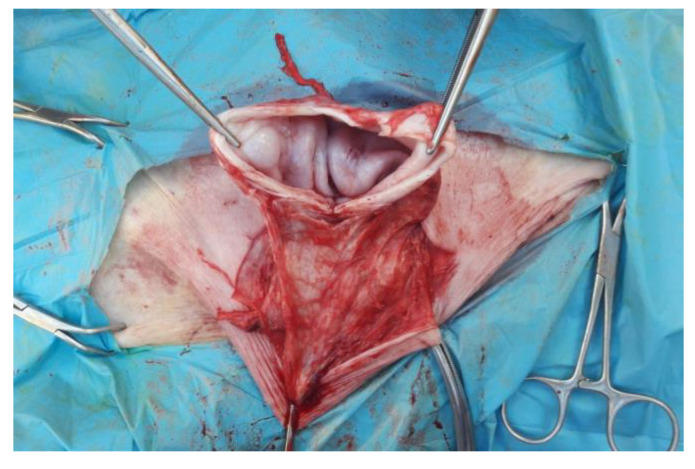
Incision of the internal hernial sac.

The skin has been incised in a half-moon shape (Figure 2); the surgery continued with the isolation of the hernial sac, with the aid of blunt scissors, or manually, with the fingers (Figure 3). The hernial sac was incised, thus highlighting the hernial ring and its contents (Figure 4). The herniated organs, when present, were repositioned in the abdomen through the hernia port, as well as the umbilical vessels after ligature.

After bleeding the sclerotic margins of the port, traditional herniorrhaphy was performed, applying horizontal “U” points, in Vicryl 0 or 1 or 2, depending on the size of the animal.

**Figure 5:**
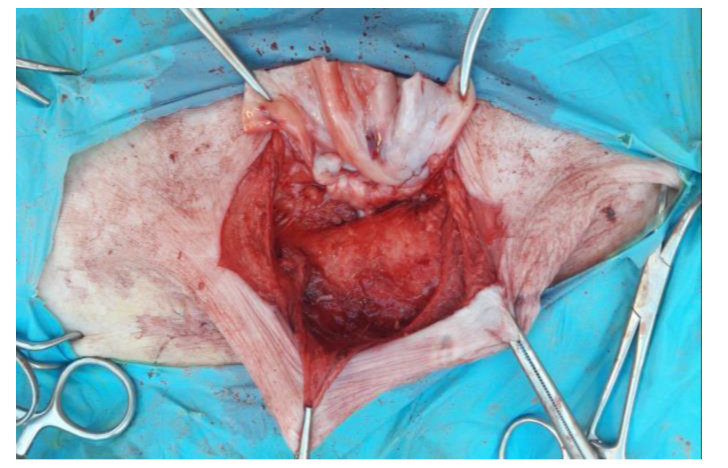
Preparation of the autologous flap by incision of the hernial sac.

**Figure 6:**
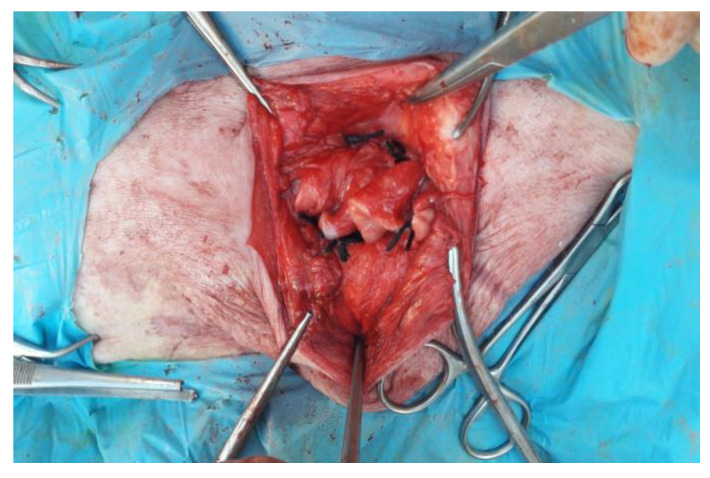
Autologous flap suture on herniorrhaphy.

Following completed herniorrhaphy, the hernial peritoneal sac was reduced and shaped (Figure 5) and positioned above the previous suture. This autologous vascular flap was then fixed with single interrupted sutures with Vicryl, in order to act as a reinforcement for the herniorrhaphy (Figure 6). The operations were concluded with a continuous suture of the subcutaneous layer to bring the margins closer, and with the suture of the skin with single interrupted sutures, applying horizontal “U” points, also in Vicryl 0 or 1.

At the end of the surgery, the animals were taken to a quiet awakening area to be kept under observation [40,44].

The animals were all subjected to general antibiotic therapy with broad-spectrum drugs by administering Amoxicillin (Betamox LA, 150 mg/mL, Vétoquinol, Bertinoro, Italy), at a dosage of 1 mL/10 kg by deep intramuscular route, for 3–5 days; the wound was medicated immediately after surgery and treated with an antibiotic spray (Terramicina Spray, Zoetis, Roma, Italy), applied twice a day for 5 days.

## 3. Results

In all swine examined, the umbilical lesions showed swellings ranging in size from 5 to 10 cm of generally rounded shape and smooth surface, with a fibrous and regular hernial ring. The lesions were neither hot nor painful, except for one subject. In the latter, in fact, present in the hernial contents of some parts of the intestine with circulation problems, there was a slight degree of pain on palpation. In all cases described it was possible to reduce the swellings through palpation.

During surgery, the herniated organs were found to be vital without necrotic alterations in all the operated swine; they have been detached from the adhesions and appropriately repositioned.

Three follow-ups were carried out: 7 days after surgery; 30 days after surgery; 60 days after surgery. Short-term and long-term follow-ups included regular checks on patients undergoing surgery.

In particular, in the first follow-up carried out a week after surgery, the surgical wounds were dry and free of infections.

One month after the surgery, the second follow-up allowed to highlight dry and healed wounds and some sutures had fallen out.

After 2 months, the third follow-up of the patients showed the fall and/or resorption of all sutures; no wound dehiscence had occurred, and the scars were evident. Therefore, the third follow-up allowed us to detect the complete healing of the surgical incision with the functional recovery of the herniated organs and an optimal get back of the general conditions of the subjects.

In all the operated cases, the success was complete: the sutures were perfect at 7 days and 30 days with rapid healing without the presence of postoperative infections, apart from a single case in which the formation of a modest serous effusion occurred, which was drained. No fixed drainage was applied in any of the subjects operated. There was no relapse after two months and the postoperative course was optimal.

## 4. Discussion

An umbilical hernia is one of the most common developmental defects in swine [2]. Considering that swine with this defect represent an economic loss [1,29,45], hernia repair would not only avoid possible complications (ulcerations, abscesses, strangulation, etc.) [32,33] but would allow the affected animals to recover and be slaughtered at the ideal weight, with an adequate economic return for the farmer.

Among the conservative methods, the most concrete technique is that implemented by Pollicino in piglets, with the application of Elastrator rings on the hernial sac [27]; due to the reported relapses, in our opinion, this method does not provide a satisfactory therapeutic procedure for resolution of umbilical hernia.

Surgery is the treatment of choice in the vast majority of symptomatic or asymptomatic umbilical hernias [46,47]. Umbilical surgery can be successfully performed in swine with deep sedation and local or general anesthesia. The method chosen depends mainly on the operator’s preference, considering the size and temperament of the animal and the difficulty expected for the intervention [38,39,40,41,43,44,46,48].

As far as the hernia is concerned, the open technique is more used than the closed one as very often umbilical hernias reach large dimensions or have complications that require the opening of the hernial sac.

However, as shown by a study performed on 34 calves affected by umbilical hernia, in which the two different methods of herniorrhaphy are compared, the complications of hernia are more frequent with the open method, also showing a higher percentage of recurrence [12].

The same study also showed that closed herniorrhaphy ensured shorter recovery times and an excellent cure rate so these results suggest that the closed technique is better than the open method, although this is valid for the surgical treatment of simple, small and reducible hernias [12].

In the case of umbilical hernias of large dimensions or complicated by adhesions, in which the surgical opening is such as not to allow closure according to the traditional method, many authors prefer to perform hernioplasty through the implantation of resorbable synthetic material that is affixed to the suture of the hernial port in order to perform a suture without tension and reinforce the latter [49], improving scarring and reducing recurrences [34].

The technique implemented by Gnemmi et al., in the treatment of umbilical hernia in cattle, in which an alloplastic membrane of Gore-Tex was used as a prosthesis, is in our opinion useful every time you have to do umbilical hernias in animals weighing more than 150 kg but above all for the surgical resolution of omphalitis which requires the surgical removal of a large necrotic area around the umbilical ring. We recognize that the Gore-Tex membranes are perfectly histocompatible and allow a successful intervention with rapid healing without postoperative infections or relapses [23].

In calves, polypropylene nets applied to the hernial ring after the reduction of the hernia were also used as prostheses [34]. This material has the advantage of being resistant and perfectly incorporated into the fabric. However, it has the defect of causing excessive adhesion to the abdominal muscles and underlying viscera [50]. This problem could be overcome by positioning the polypropylene net in the outermost part of the abdominal wall, in correspondence with the subcutaneous tissue [34] but, despite this, some subjects treated showed inflammatory edema at the level of the umbilical region in how much the polypropylene net, still representing a foreign body, caused inflammation resulting in treatment with antibiotics and NSAIDs for two weeks. The limit of these techniques lies in the high cost of the Gore-Tex and polypropylene membranes, certainly not feasible in the surgery of livestock.

Synthetic materials in hernioplasty are nowadays mostly replaced by the use of biological materials such as fibrin hydrogel, alginate, chitosan, hyaluronic acid and acellular collagen grafts. The excellent biocompatibility, biodegradability and low antigenicity make collagen one of the most useful biomaterials [37].

The effectiveness of the use of biological materials has been demonstrated by some authors in India, who have practiced interesting hernioplasty interventions in a group of swine affected by umbilical hernia using prostheses consisting of acellularized buffalo diaphragm, with different concentrations of sodium deoxycholate. These prostheses, in addition to not causing any rejection, have allowed a complete recovery of all animals, demonstrating the safety of their use for umbilical hernia of swine [51]. However, we believe that the limitations of this technique are due to the need for further research to verify the immunogenic properties of this material after xenogenic transplantation in various sites.

Therefore, recognizing the value of alloplastic prostheses in the success of the intervention and considering that, in the medical literature, it is shown that the use of surgical meshes reduces the recurrence rate compared to repair without surgical mesh, and also taking into account the importance of characteristics of biological materials for their biocompatibility, we decided to replace the synthetic prosthetic material with an autologous prosthesis obtained by obtaining a vascularized flap from the hernial sac.

Our experience makes use of the success of the described technique, already applied for years in young cattle [40,44]. In fact, the idea of using an autologous prosthesis arose from a double consideration: the great stress to which the tissues are subjected, especially in large animals, which requires the application of a reinforcement that remains stable at least for the first 15 days of the postoperative period; the hernial sac, robust and well vascularized, provides excellent tissue compatibility and, thanks to the constant blood flow, ensures that there is always the presence of cellular defense and reaction elements, with the formation of a rigid and “adhesive” layer of fibrin.

It was therefore natural to think of exploiting these characteristics of the autologous flap in such a way as to make it perform the functions of a prosthesis, eliminating the unwanted effects of heterologous meshes, including, not of secondary importance, their prohibitive cost. These claims are substantiated by the results of literature on the use of heterologous materials to repair hernias [23,34].

To confirm this, after a period of 60 days the follow-up of all treated patients confirmed the complete healing of the lesion with functional recovery of the herniated organs and the general state of the subjects.

In our opinion, a limitation of our study is related to the number of subjects undergoing surgery. Future prospects include expanding the number of swine operated on using the technique of hernioplasty with a peritoneal flap to allow us to assess any limitations of the surgical technique, which have not yet manifested themselves.

## 5. Conclusions

Our experience has allowed us to overcome an important limitation of hernioplasty in swine, represented by the high price of alloplastic membranes which often increases the costs that farmers must face and can preclude the surgical correction of this pathology.

This surgical method has the purpose of conferring considerable resistance to the herniorrhaphy performed and therefore preventing relapses, without using synthetic prosthetic materials which, although histocompatible and non-antigenic, represent foreign bodies that make the surgical wound very sensitive to bacterial colonization.

Considering this it can be understood how the use of a flap of the hernial sac, and therefore of autologous tissue, has allowed for obtaining excellent results, allowing to have a biomaterial prosthesis with excellent tissue compatibility at no cost, able to evoke the inflammatory response allowing rapid healing of patients.

## Data Availability

Not applicable.
